# Ceftriaxone‐Resistant *Escherichia coli* Sepsis in an Infant With Spinal Muscular Atrophy Type 1 Receiving Risdiplam: A Case Report

**DOI:** 10.1002/ccr3.73118

**Published:** 2026-07-07

**Authors:** Momen Zetawi, Razan Shoman, Asem Afana, Shahd Aldarak, Ro'a Draidi, Bara AbuBaha, Raheeq Jaradat, Firas Khouli, Hossam Salameh

**Affiliations:** ^1^ Department of Medicine, Faculty of Medicine and Health Sciences An‐Najah National University Nablus Palestine; ^2^ Tulkarm Governmental Hospital Tulkarm Palestine

**Keywords:** antimicrobial resistance, case report, ceftriaxone‐resistant sepsis, *Escherichia coli*, neuromuscular weakness, pediatric sepsis, PICU, respiratory failure, risdiplam, spinal muscular atrophy type 1 (SMA type 1)

## Abstract

Even in the risdiplam era, infants with SMA type 1 remain vulnerable to severe resistant infections because respiratory and bulbar weakness persist. Early cultures, susceptibility‐guided antibiotics, airway‐clearance measures, ventilatory support, and multidisciplinary PICU care are crucial to stabilize sepsis, prevent respiratory deterioration, and improve outcomes in this fragile population overall.

## 1 Introduction

Spinal muscular atrophy (SMA) is a rare autosomal recessive neuromuscular disorder caused by biallelic deletions or pathogenic variants in the survival motor neuron 1 (SMN1) gene, resulting in deficiency of survival motor neuron protein and progressive degeneration of anterior horn motor neurons. Clinically, this leads to generalized muscle weakness, hypotonia, and respiratory compromise [1]. SMA is classified into subtypes (0–4) based on age of onset and maximal motor function achieved, with disease severity partly influenced by SMN2 copy number [1, 2].

SMA type 1 (Werdnig–Hoffmann disease) is the most severe and common phenotype, accounting for approximately 60% of SMA cases [3]. It typically presents within the first 6 months of life with profound hypotonia, bulbar dysfunction, and progressive respiratory muscle weakness. Historically, mortality exceeded 90% within the first 2 years of life, most commonly due to respiratory failure [3].

The introduction of disease‐modifying therapies such as nusinersen, onasemnogene abeparvovec, and risdiplam has significantly changed the natural history of SMA [4]. Risdiplam is an orally administered SMN2 splicing modifier that increases production of functional SMN protein in both central and peripheral tissues [5]. Clinical trials demonstrated improved survival and motor milestone achievement compared with historical natural history cohorts [5].

Despite these advances, infants with SMA type 1 remain medically fragile due to respiratory muscle weakness, impaired airway clearance, bulbar dysfunction, and frequent healthcare exposure [2, 3, 6]. Consequently, severe infections and sepsis remain an important cause of morbidity in this population. *Escherichia coli* is among the most common pathogens causing infant sepsis, and antimicrobial resistance may complicate management and delay effective therapy.

We report a case of ceftriaxone‐resistant *Escherichia coli* sepsis in an infant with SMA type 1 receiving risdiplam, highlighting infectious vulnerability in the disease‐modifying therapy era.

## 2 Case History/Examination

A 4‐month‐old female infant with genetically confirmed spinal muscular atrophy type 1, receiving oral risdiplam therapy, presented with progressive tachypnea and increased work of breathing without documented fever. Oxygen saturation was 89% on room air, and temperature was 37.5°C.

Risdiplam was initiated when the patient was approximately 4 months and 25 days old. At treatment initiation, the child was markedly symptomatic from SMA, having been hospitalized for respiratory distress and left‐sided pneumonia. She demonstrated severe hypotonia and respiratory compromise requiring oxygen supplementation initially and subsequently invasive mechanical ventilation. At the time risdiplam was started, she remained ventilator‐dependent (PEEP 6 cmH_2_O, FiO_2_ 35%), with persistent mild respiratory distress and retractions and required nasogastric tube feeding.

On examination, the infant appeared in moderate respiratory distress with subcostal retractions. Chest examination revealed decreased air entry over the left lung field with bilateral crepitations, raising suspicion of pneumonia. Neurological examination demonstrated generalized hypotonia consistent with SMA. The anterior fontanel was open and flat, and abdominal examination was soft with no hepatosplenomegaly.

## 3 Differential Diagnosis, Investigations and Treatment

### 3.1 Differential Diagnosis

The initial differential diagnosis included severe bacterial pneumonia, sepsis secondary to urinary tract infection, and aspiration‐related respiratory infection, given the infant's respiratory distress and underlying neuromuscular disease. Infants with SMA are particularly susceptible to respiratory infections because respiratory muscle weakness impairs airway clearance and cough effectiveness.

### 3.2 Investigations

Laboratory investigations shown in Table 1 demonstrated leukocytosis and markedly elevated inflammatory markers, while arterial blood gas analysis revealed hypoxemia. Blood cultures grew *Escherichia coli* resistant to ceftriaxone but sensitive to piperacillin–tazobactam, confirming Gram‐negative sepsis. Urine culture also yielded *E. coli* , suggesting a systemic infectious process. These microbiological findings were essential in guiding targeted antimicrobial therapy.

**TABLE 1:** Initial laboratory investigations.

Investigation	Result	Reference range	Interpretation
White blood cell count (WBC)	16.2 × 10^3^/μL	5–15 × 10^3^/μL	Leukocytosis
C‐reactive protein (CRP)	121.7 mg/L	< 5 mg/L	Markedly elevated
Fasting blood glucose	112 mg/dL	70–110 mg/dL	Within normal limits
Arterial partial pressure of oxygen (PaO_2_)	62.8 mmHg	80–100 mmHg	Hypoxemia

### 3.3 Treatment

**FIGURE 1:**
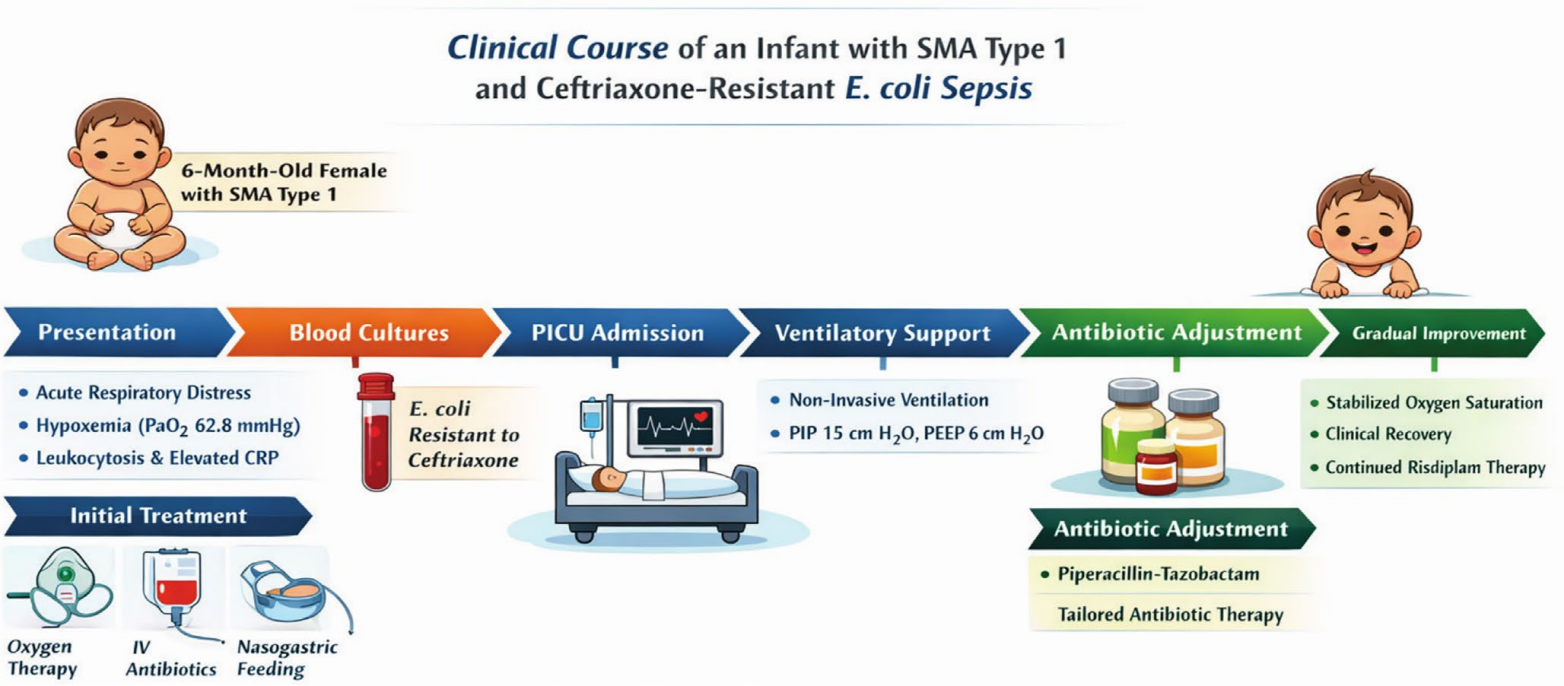
Visual timeline of the clinical course.

The patient was admitted to the pediatric intensive care unit (PICU) for close monitoring and isolation. Management included supplemental oxygen via face mask, intravenous fluid resuscitation, careful nasogastric feeding, chest physiotherapy, airway suctioning, and nebulization with hypertonic saline and ipratropium. Empirical antibiotic therapy was initiated and later escalated to piperacillin–tazobactam according to culture sensitivity results. Due to progressive respiratory compromise, non‐invasive ventilatory support was initiated (PIP 15 cmH_2_O, PEEP 6 cmH_2_O, FiO_2_ 35%). Risdiplam therapy was continued during hospitalization, and summarization of the clinical course is demonstrated in Figure 1.

## 4 Conclusion and Results (Outcome and Follow‐Up)

Following targeted antimicrobial therapy and intensive supportive care, the patient showed gradual respiratory improvement and stabilization of oxygen saturation and vital signs. Adequate urine output and tolerance of nasogastric feeding were maintained, and inflammatory markers gradually improved. This case illustrates that infants with SMA type 1 remain highly susceptible to severe infections despite disease‐modifying therapy, and highlights the importance of early microbiological diagnosis, prompt targeted antibiotic therapy, and multidisciplinary intensive care management.

## 5 Discussion

Spinal muscular atrophy type 1 represents the most severe phenotype of SMA and is characterized by profound hypotonia, bulbar dysfunction, and progressive respiratory muscle weakness, which predispose infants to respiratory infections due to impaired airway clearance and ineffective cough [1, 7].

In the present case, the infant developed ceftriaxone‐resistant *Escherichia coli* sepsis, requiring intensive care and ventilatory support. Antimicrobial resistance is increasingly recognized in pediatric intensive care units and can delay effective therapy when empirical antibiotics are inadequate [4, 5]. Early blood culture acquisition and susceptibility testing are therefore essential for guiding targeted antimicrobial therapy.

Respiratory complications in SMA often reflect underlying neuromuscular weakness rather than reversible pulmonary disease alone. Weakness of respiratory muscles and bulbar dysfunction impair spontaneous breathing and airway protection, frequently requiring prolonged respiratory support and airway clearance interventions [1, 7].

Risdiplam has significantly improved survival and motor outcomes in infants with SMA type 1 by increasing SMN protein production [8, 9]. However, respiratory infections remain common in this population, reflecting the intrinsic vulnerability associated with severe neuromuscular disease rather than direct drug‐related immunosuppression [10].

Treatment of SMA using risdiplam has been proven to improve motor function in patients. Better prognosis is observed with earlier initiation of treatment and overall more functional patient at baseline status. But no significant improvement in nutritional and respiratory function was reported [11].

The provided treatment for SMA targets genetic causes of the disease and increases the SMN protein. But the medical influence of the absent protein may not simply be solved, explaining why there is respiratory function less likely to completely recover [12].

Drug‐drug interactions of risdiplam administrator with midazolam showed that midazolam level increased by 11% in vivo, which is 18 times lower than in vitro drug levels, suggesting that CYP3A inactivation is exaggerated in vitro. The enzyme inhibition occurred in the human intestine rather than liver. So no significant drug interaction is detected [13].

Midazolam wasn't provided in our patient, so there is no direct link between the ineffectiveness of risdiplam in improving respiratory outcome and drug–drug interactions.

This case highlights the complex interplay between neuromuscular weakness, antimicrobial resistance, and critical care management in infants with SMA. Despite advances in disease‐modifying therapies, severe bacterial infections remain a major clinical threat in this medically fragile population [14, 15].

## Author Contributions

**Momen Zetawi:** supervision, writing – original draft. **Shahd Aldarak:** writing – original draft. **Ro'a Draidi:** writing – original draft. **Bara AbuBaha:** resources. **Raheeq Jaradat:** writing – original draft. **Firas Khouli:** resources. **Razan Shoman:** writing – review and editing. **Asem Afana:** writing – review and editing. **Hossam Salameh:** supervision.

## Funding

The authors have nothing to report.

## Ethics Statement

Compliance with ethical standards. All procedures performed in this report involving human participants were in accordance with the ethical standards of the institutional, national research committee, and with the 1964 Helsinki declaration and its later amendments or comparable ethical standards.

## Consent

The authors obtained verbal and written informed consent from the patient regarding this case and any accompanying images. A copy of the written consent is available for review by the Editor‐in‐Chief of this journal on request.

The institution to which this case was admitted does not require approval for writing this case report.

## Conflicts of Interest

The authors declare no conflicts of interest.

## Data Availability

Data will be provided on request from the editor‐in‐chief due to limitations.
